# Regulation of the Membrane Insertion and Conductance Activity of the Metamorphic Chloride Intracellular Channel Protein CLIC1 by Cholesterol

**DOI:** 10.1371/journal.pone.0056948

**Published:** 2013-02-14

**Authors:** Stella M. Valenzuela, Heba Alkhamici, Louise J. Brown, Oscar C. Almond, Sophia C. Goodchild, Sonia Carne, Paul M. G. Curmi, Stephen A. Holt, Bruce A. Cornell

**Affiliations:** 1 School of Medical and Molecular Biosciences, University of Technology Sydney, Sydney, New South Wales, Australia; 2 Department of Chemistry and Biomolecular Sciences, Macquarie University, Sydney, New South Wales, Australia; 3 Surgical Diagnostics Pty Ltd, Sydney, New South Wales, Australia; 4 School of Physics, University of New South Wales, Sydney, New South Wales, Australia; 5 Centre for Applied Medical Research, St Vincent's Hospital, Sydney, New South Wales, Australia; 6 Bragg Institute, Australian Nuclear Science and Technology Organisation, Sydney, New South Wales, Australia; University of Cambridge, United Kingdom

## Abstract

The Chloride Intracellular ion channel protein CLIC1 has the ability to spontaneously insert into lipid membranes from a soluble, globular state. The precise mechanism of how this occurs and what regulates this insertion is still largely unknown, although factors such as pH and redox environment are known contributors. In the current study, we demonstrate that the presence and concentration of cholesterol in the membrane regulates the spontaneous insertion of CLIC1 into the membrane as well as its ion channel activity. The study employed pressure versus area change measurements of Langmuir lipid monolayer films; and impedance spectroscopy measurements using tethered bilayer membranes to monitor membrane conductance during and following the addition of CLIC1 protein. The observed cholesterol dependent behaviour of CLIC1 is highly reminiscent of the cholesterol-dependent-cytolysin family of bacterial pore-forming proteins, suggesting common regulatory mechanisms for spontaneous protein insertion into the membrane bilayer.

## Introduction

Non-classical integral membrane proteins such as members of the Chloride Intracellular Ion Channel (CLIC) family of proteins challenge our traditional assumptions regarding the processes by which such proteins insert into membranes. In general, classical integral membrane proteins are synthesized in cells by ribosomes that are directed to the endoplasmic reticulum (ER) via signal sequences located within the protein itself. The transmembrane domain(s) remain lodged in the ER membrane in preparation for eventual delivery of the processed proteins to their final destination, either the cell plasma membrane or another intracellular membrane structure.

The CLIC family proteins do not contain a signal sequence or any obvious membrane spanning domains, but are instead capable of spontaneous insertion into lipid bilayer membranes from their soluble form, and thus by-pass the traditional route of integral membrane synthesis and processing. One of their functions when located in the membrane is to act as ion channels. This has now been demonstrated *in situ* [1], [2], as well as using artificial lipid membrane systems for a number of the member proteins including, CLICs 1, 2, 4, 5 [3], [4], [5], [6], [7], [8], [9], [10], [11], [12]. Channel activity has also been demonstrated for the invertebrate orthologues, EXC-4 from *Caenorhabditis elegans* and *Dm*-CLIC from *Drosophila melanogaster* [13].

The human CLIC1 member of the CLIC family has an additional distinct property where its soluble form exists in two stable conformations. The transition between the two soluble states arises via a large rearrangement of the CLIC1 N-terminal domain under the influence of oxidation[7]. Because of this, it has been included into the recently classed family of “metamorphic” proteins[14]. It is postulated that rearrangement of the highly ‘plastic’ N-terminal domain is necessary for the protein's ability to auto-insert into a membrane environment [7], [15], [16], [17], [18].

There are in fact a number of soluble human proteins which are also known to spontaneously insert and self-assemble into lipid membranes in the absence of cellular machinery. One such group is the annexin family of proteins[19]. This family of proteins spontaneously bind to membranes in response to environmental cues such as changes in intracellular calcium concentrations [19] or, in the case of annexin B12, reversibly insert into membranes at acidic pH [20]. The apoptotic soluble Bax protein has also been shown by atomic force microscopy and liposome leakage studies to spontaneously insert into membranes and form pores, enhanced by the presence of calcium[21], [22].

The process of spontaneous membrane insertion is also known to occur for a number of bacterial toxins and colicins, such as the 293-residue polypeptide, α-hemolysin [23]. α-hemolysin is secreted by *Staphylococcus aureus* as a water-soluble monomer which can bind to and form heptameric pores in lipid bilayers [24], [25]. Artificial truncated protein constructs have also been used to study these processes of membrane auto-insertion [26]. The artificial membrane peptide pH (low) insertion peptide (pHLIP) is a 36-amino acid peptide containing the sequence of the C-helix of the integral membrane protein bacteriorhodopsin. pHILP has been described as ‘living in three worlds’:- unstructured but soluble in near neutral aqueous solution; binding to the surface of lipid bilayers as an extended chain; and, as a transmembrane α-helix in lipid membranes for which insertion is triggered by low pH [26].

Factors known to dramatically influence the interaction of proteins with the membrane architecture include phospholipid composition and membrane cholesterol. The cholesterol dependent-cytolysins (CDCs) are a large family of pore-forming proteins, being principally proteins from different species of Gram positive bacteria (examples: listeriolysin, perfringolysin, streptolysin and pneumolysin) but also include the human proteins perforin and the complement membrane attack complex [27]. These proteins share the common feature of interacting with membranes via a two-step process, of which the first involves binding to cholesterol within the membrane followed by insertion [27], [28]. The interaction of the protein CLIC1 with membranes has also been found to be lipid dependent, with studies showing that different combinations of phospholipids and cholesterol result in different functional activity of the protein [8], [11]. One of these studies demonstrated that increasing cholesterol from 10% to 30% of the lipid content in a liposome chloride efflux assay resulted in a decrease of CLIC1 functional activity [11].

In the present study, we investigated the ability of the membrane auto-inserting proteins α-hemolysin, listeriolysin-O and CLIC1 to form conductive channels in a tethered lipid bilayer system. The functional ion channel activity of these proteins was assessed using impedance spectroscopy, where changes in the bilayer conductance demonstrates the proteins' functional activity upon insertion. We specifically investigated the influence of varying concentrations of cholesterol in the membrane, on the ability of CLIC1 to form conductive channels in the tethered bilayer membranes. In addition, Langmuir monolayer film experiments confirmed the importance of cholesterol in order for CLIC1 to auto-insert into a membrane.

## Materials and Methods

α-Hemolysin was purchased from Sigma Aldrich, Australia; Listeriolysin was purchased from Sapphire, Australia. The monolayer films consisted of 1-palmitoyl-2-oleoyl-sn-glycero-3-phosphocholine (POPC) (Avanti Polar Lipids, USA) and Cholesterol (Sigma, Australia).

### Recombinant CLIC1 Protein Expression and Purification

Recombinant CLIC1 protein was expressed in the *E-coli* bacterial strain, BL21(DE3) using the His-tag pET28a vector system (Novagen), as previously described [15]. Briefly, transformed cells were grown in 2xYT media at 37 °C in a shaking incubator, with CLIC1 protein expression induced following addition of 1 mM isopropylthio-beta-galactoside (IPTG) at mid-log growth phase. The cell culture was then allowed to grow for a further 16 h at 20 °C before lysis. The soluble lysate was then run through a Ni-NTA chromatography column (Novagen) followed by cleavage and release of the protein from its His-tag using 50 NIH units of bovine plasma thrombin (Sigma Aldrich) per litre of cell culture. CLIC1 protein was then incubated with 0.5 mM TCEP followed by further purification by size exclusion chromatography Superdex-75 prep grade high performance chromatography column (GE Healthcare, Piscataway, USA). The column was initially equilibrated in column sizing buffer (100 mM KCl, 0.5 mM TCEP, 1 mM NaN_3_, 20 mM HEPES pH 7.5). Protein purity was confirmed using SDS-PAGE analysis and had an estimated purity of 99%. All purified CLIC1 protein consisted of the CLIC1 sequence with an extra three residues at the N-terminus (Gly-Ser-His) as a result of the thrombin cleavage site in the fusion construct.

### Preparation of tethered membranes and impedance spectroscopy

Artificial lipid membranes were formed first by using lipid coated gold electrodes that contain a first layer of 10% tethered phytanyl bis-tetraethyleneglycol benzyldisulphide and hydroxyterminated-bis-tetra-ethyleneglycol lipids from (Surgical Diagnostics Pty, Ltd). This first layer of lipids was then rinsed with 8 µL of a 7:3 molar ratio of Diphytanyletherphosphatidylcholine and Glycerodiphytanylether (Surgical Diagnostics Pty, Ltd) in ethanol, with or without cholesterol (Sigma) of a molar percentage (mol%) ranging between 0 – 50 mol% relative to the phospholipid content. {A 50 mol% cholesterol level represents a membrane containing approximately 30% cholesterol by weight. Most mammalian cells have a typical cholesterol composition of 20% cholesterol by weight in their plasma membranes[29].} This was then followed by addition of aqueous Hepes/KCl buffer (0.1 M KCl, 0.1 mM HEPES and 0.01 mM CaCl_2_ of pH 6.5) which drives the self-assembly of the bilayer membrane. The tethered bilayer lipid membranes were then further rinsed with the Hepes/KCL buffer containing 0.5 mM TCEP with the pH re-adjusted to 6.5. Membranes were allowed to stabilize for ∼1 hour before proteins were added in a final volume of 100 µL at concentrations between 7.4 – 14.8 µM of CLIC1 in Hepes/KCl containing 0.5 mM TCEP (pH 6.5), 10 – 400 nM α-Hemolysin in Hepes/KCl buffer and Listeriolysin-O 2 µM in Hepes/KCl buffer. The change in conductance across the lipid bilayer membranes in response to the formation of conductive channels by the proteins was measured by impedance spectroscopy as previously described [30].

### Pre-incubation of CLIC1 with cholesterol

CLIC1 protein (20 µg/100 µL buffer) was incubated with 2 µL of cholesterol dissolved in ethanol (13.3 mg cholesterol per mL of ethanol) for 1 hour on ice prior to addition of the protein sample to the tethered membranes.

### Langmuir films

POPC lipid and cholesterol were each dissolved in spectroscopic grade chloroform (Sigma) at a concentration of 1 mg/mL. Lipid films were prepared by mixing these solutions to produce a final solution consisting of a 5:1 POPC:cholesterol molar ratio. The Langmuir trough (Nima Technology), area 105 cm^2^ and thermostatted at 25 °C, was thoroughly cleaned and rinsed with MilliQ water prior to use. Prior to each experiment the trough was filled with 30 mL buffer solution and checked for cleanliness. Typically 10–20 µL of POPC or POPC:cholesterol solution was added drop wise to the buffer surface. The chloroform was allowed to evaporate for 5–10 minutes and then a compression cycle at 10 cm^2^/minute was performed to confirm the expected behaviour of the film. The monolayer was then compressed to a pressure of 20 mN/m and held under pressure control. The surface area was then controlled via a feedback loop to maintain the required surface pressure. In a second series of experiments the monolayer was allowed to equilibrate at 20 mN/m for 5 minutes, CLIC1 was then added to the aqueous subphase (final 0.0363 µM) under the lipid film. The pressure and area were then recorded as a function of time for all experiments. To allow comparison of separate experiments, where the initial trough area may have been different, the change in area is presented as a percentage change from the initial value.

## Results and Discussion

**Figure 1:**
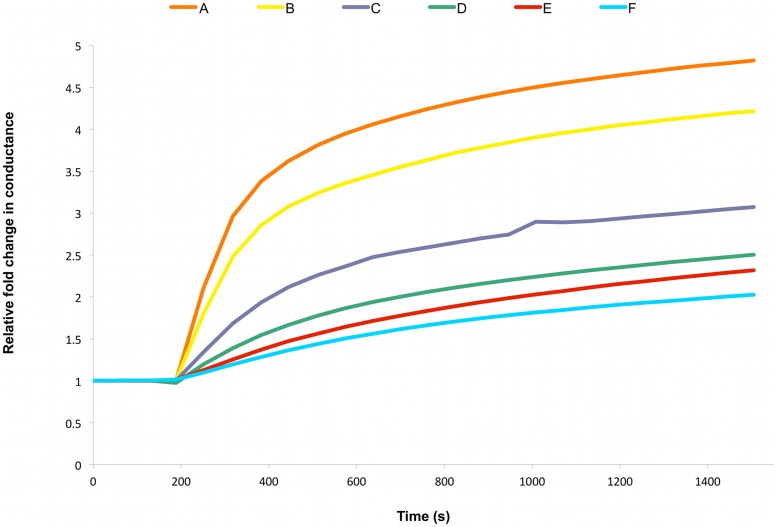
Membrane Conductance of α-hemolysin. Representative impedance spectroscopy recordings of 50 nM α-hemolysin added to tethered membranes made from AM199 lipid containing varying amounts of cholesterol (mol% chol) (A) 0, (B) 10, (C) 20, (D) 30, (E) 40, (F) 50; [n = 3].

In order to assess the membrane insertion and ion channel activity of a group of spontaneously inserting membrane proteins, impedance spectroscopy analysis of a tethered bilayer membrane on a gold substrate [30] was employed. The impedance spectroscopy allows for the determination of the membrane capacitance and conductance. The effect of the membrane composition – specifically cholesterol concentration – on the protein insertion and channel activity was characterised. Addition of equal amounts of the protein α-hemolysin to membranes containing varying amounts of cholesterol (0–50 mol%) clearly demonstrated an increase in membrane conductance due to α-hemolysin. Although α-hemolysin is not classed as a member of the cholesterol-dependent-cytolysins, recent studies in the literature indicate cholesterol does impact upon its activity and membrane interactions [31], with depletion of cholesterol from membranes resulting in arrest in the assembly of the α-hemolysin [32]. In the current study however, the highest levels of membrane conductance due to α-hemolysin were observed in the sample containing no cholesterol in the membrane (Figure 1). It is interesting to note, that as the level of cholesterol in the membrane was increased, membrane conductance by α-hemolysin was seen to decrease in a concentration dependent manner. This suggests that the protein conductance and/or its membrane insertion were affected by the concentration of cholesterol in the membrane. The membrane spanning form of α-hemolysin is known to be a heptameric homomultimer [33]. It is conceivable that the decrease in membrane fluidity and permeability produced by increasing cholesterol levels in the membrane, results in the decreased α-hemolysin conductance. This could arise from either a decreased ability of the α-hemolysin to initially insert into the membrane, or an inability of inserted α-hemolysin to properly convert to a mature channel form.

**Figure 2:**
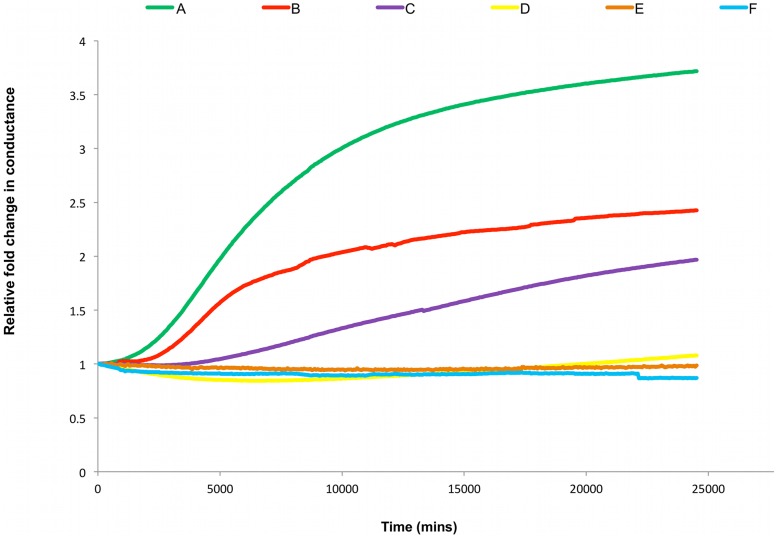
Membrane Conductance of Lysteriolsin. Representative impedance spectroscopy recordings of (A-E) 2 µM Lysteriolysin and (F) buffer control, added to tethered membranes made from AM199 containing varying amounts of cholesterol (mol% chol) (A) 50, (B) 25, (C) 12.5, (D) 6.25, (E) 0, (F) 50; [n = 3].

The second protein characterised was the spontaneously membrane inserting protein lysteriolysin-O. This protein is a member of the cholesterol-dependent-cytolysin family derived from Gram-positive bacteria, and as such, we would expect that its membrane interactions would be regulated by membrane cholesterol levels [27]. As is seen in Figure 2, in the complete absence or at low concentrations of cholesterol in the membrane (<∼6 mol%), addition of 2 µM lysteriolysin-O did not result in a change in membrane conductance. However, when an equal amount of lysteriolysin was added to membranes containing ≥12.5 mol% cholesterol, an increase in the membrane conductance was clearly noted, with highest conductance observed at 50 mol% cholesterol. It has been noted in the literature that the role of cholesterol in the CDC protein membrane interactions is not simply to act as a receptor-binding site but may in some cases be acting as a trigger for conversion of the proteins from their pre-pore to their final integral membrane pore state [34]. Studies have also shown that there exists a narrow range of cholesterol concentration between which optimal CDC binding takes place. As recently reviewed by Tweten (2005) [35] the CDCs tetanolysin and perfringolysin O show little to no binding to liposomes containing 40 mol% cholesterol, whereas at 50–55 mol% they both exhibit maximal binding. Thus it is suggested that interactions of the CDCs with membranes is occurring via cholesterol-rich rafts [35].

**Figure 3:**
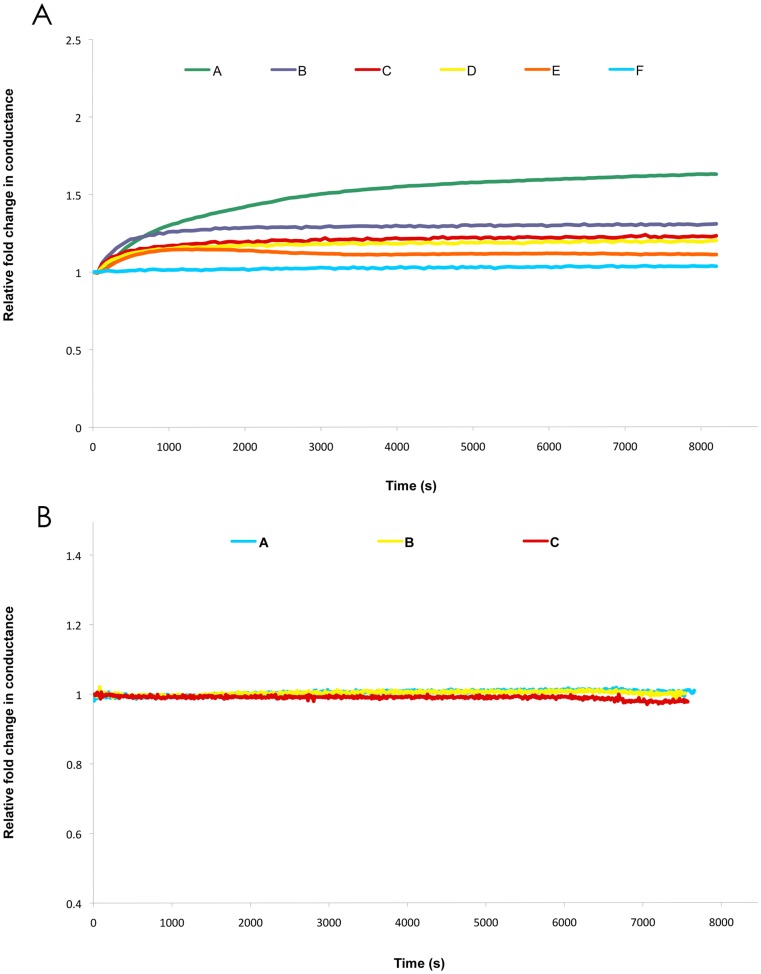
Membrane Conductance of CLIC1. **A.** Representative impedance spectroscopy recordings of (A-E) 7.4 µM CLIC1 and (F) buffer control, added to tethered membranes made from AM199 lipid containing varying amounts of cholesterol (mol% chol) (A) 50, (B) 25, (C) 12.5, (D) 6.25, (E) 0, (F) 50; [n = 3]. **B.** Representative impedance spectroscopy recordings of (A) buffer control, (B) 7.4 µM boiled CLIC1 and (C) 14.8 µM boiled CLIC1, added to tethered membranes made from AM199-PC lipid containing 25 mol% cholesterol; [n = 3].

The human protein CLIC1 has previously been shown to spontaneously insert into lipid membranes via a number of methods including tip-dip electrophysiology [12], planar lipid membranes (BLM) [8] and chloride-efflux studies [7]. In our current study using a tethered lipid bilayer membrane system, we also demonstrate that addition of soluble CLIC1 protein causes increased membrane conductance and as such, membrane insertion is subsequently inferred. As seen in Figure 3A, addition of equal amounts of CLIC1 protein to tethered membranes containing varying amounts of cholesterol (0–50 mol%), a cholesterol-dependent response was observed. Maximum conductance by CLIC1 protein was observed in membranes containing 50 mol% cholesterol (50 mol% represented the highest molar percentage of cholesterol tested), while in the control wells (membranes with 50 mol% cholesterol and no added CLIC1 protein), the membranes remained tightly sealed. At lower percentages of cholesterol in the membranes of 25, 12.5 and 6.25 mol% as well as in those containing no cholesterol, increased membrane conductance upon addition of CLIC1 protein was also observed, however the conductance levels were found to be relatively lower, compared to the membranes containing 50 mol% cholesterol. A negative control sample of boiled, denatured CLIC1 protein added to tethered membranes was found to cause no conductance changes in membranes with or without cholesterol, as seen in Figure 3B.

The relatively lower CLIC1 conductance in membranes containing ≤25 mol% cholesterol compared to 50 mol% cholesterol, holds similarities to the cholesterol concentration effect described above for the CDC proteins. In addition, the ability of CLIC1 to conduct in the absence of cholesterol, albeit at lower levels, suggests the existence of two types of protein-membrane interactions. The first being a cholesterol-independent event, which gives rise to a certain level of CLIC1 conductance; with the second being a cholesterol-dependent event, that results in a much larger CLIC1 conductance. In a former tip-dip electrophysiological study of CLIC1 conductance using pure PC lipid bilayers, the occurrence of two different ionic conductances was described [12]. The first was seen upon addition of CLIC1 protein to the external bath solution, where there was a variable lag period of null events followed by an initial small conductance channel of slow kinetics (SCSK), with multiple current levels. Over time, these events disappeared and were replaced by channels that were characterised as high conductance with fast kinetics (HCFK). It was proposed that the SCSK openings are from proteins which have not yet assembled into the final state of the multimeric HCFK CLIC1 channel [12]. These previously described ionic conductance states of CLIC1, when considered in conjunction with our current findings, are suggestive that cholesterol may act in a manner which facilitates the quaternary assembly of CLIC1 within the membrane. This however is speculative and further investigations are needed to determine whether a cholesterol-dependent effect on CLIC1 oligomerisation/quaternary assembly within the membrane exists.

**Figure 4:**
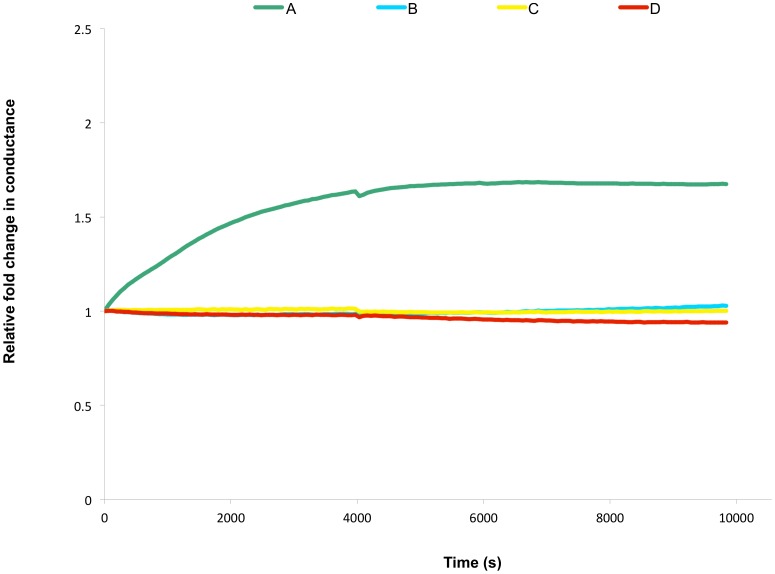
Effects of Cholesterol on the Membrane Conductance of CLIC1. Representative impedance spectroscopy recordings of (A) 7.4 µM CLIC1 (in 100 µL volume) pre-incubated with 2 µL ethanol (solvent control), (B) no protein added (buffer control), (C) 7.4 µM CLIC1 (in 100 µL volume) pre-incubated with 2 µL cholesterol in EtOH, and (D) 14.8 µM CLIC1 (in 100 µL volume) pre-incubated with 2 µL cholesterol in EtOH. Samples added to tethered membranes made from AM199 lipid containing 50 mol% cholesterol; [n = 3].

In an attempt to further define the role of cholesterol in regulating CLIC1's activity as a conductive ion channel, an experiment was carried out which involved pre-incubation of the CLIC1 protein with cholesterol prior to its addition to tethered membranes. This approach had previously been used by others who had shown that pre-incubation with cholesterol inhibited the cytolytic activity of the CDCs, by preventing them from entering the membrane and creating a conductive pore [35]. As seen in Figure 4, pre-incubation of CLIC1 protein with cholesterol prior to its addition to membranes, resulted in complete abrogation of CLIC1 conductance in membranes containing 50 mol% cholesterol. An ethanol carrier control was also included which showed no effect on CLIC1's ability to increase membrane conductance (Figure 4). These results therefore suggest that cholesterol is acting as a potential binding or docking site on the membrane to which the CLIC1 attaches and subsequently inserts into the membrane and/or is required for the subsequent quaternary structural assembly of the protein, once associated with the membrane. These are similar mechanisms to the ones known to exist for the CDCs in which cholesterol appears to act either as the membrane binding site for some of the CDCs, but also has an additional role in the conversion of the CDC pre-pore to final pore complex in membranes [34]. Elucidation of the precise role of cholesterol in regulating CLIC1 membrane interactions and ion channel activity remains.

**Figure 5:**
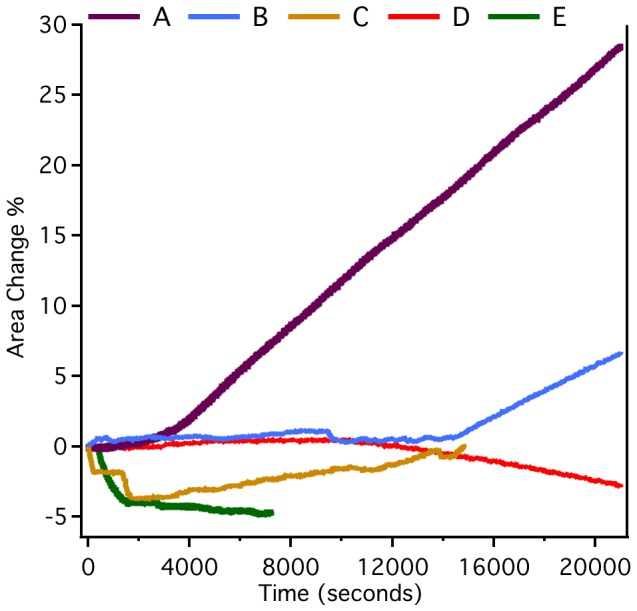
Insertion of CLIC1 into Lipid Monolayers. Representative area traces of Langmuir films held at a surface pressure of 20 mN/m with 0.036 µM CLIC1 (A-C) and (D, E) no CLIC, added beneath POPC monolayers containing cholesterol (mol% chol) (A) 16.7, (B) 9.0, (C) 0.0, (D) 16.7, (E) 0.

Langmuir monolayer film studies using POPC lipid with and without cholesterol, also confirmed the regulatory effect of cholesterol on CLIC1 membrane interactions. CLIC1 protein insertion and/or membrane interactions were clearly seen to be reduced in membranes where cholesterol was absent from the monolayer. Figure 5 shows the % change in area traces for a number of different monolayer conditions. It was noted that in the films with no cholesterol and where no CLIC1 protein was added, there was an initial relaxation or decrease in the film layer, followed by relative stability in the film over a number of hours (Figure 5, E). For the POPC monolayers lacking cholesterol, addition of CLIC1 saw less initial relaxation of the membrane, but there was also no obvious overall increase in the % change in area compared to the initial trough area suggesting some phospholipid headgroup and protein interactions, but little to no protein insertion into the membrane monolayer (Figure 5, C). In the films containing 16.7 mol% cholesterol, there was a large increase in trough area over time, following CLIC1 protein addition, clearly indicating CLIC1 protein insertion into the monolayer (Figure 5, A). Reducing the cholesterol content of the monolayer to 9 mol% reduced the extent of protein insertion over the same time period compared to 16.7 mol% cholesterol, and also increased the lag time prior for commencement of insertion (Figure 5, B). Finally a lipid film containing 16.7 mol% cholesterol but no added CLIC1 was more stable than pure POPC before finally showing a small area decrease (Figure 5, D).

Taken together, the impedance spectroscopy and Langmuir film studies clearly demonstrate a direct relationship between cholesterol concentrations in membranes and the ability of the protein CLIC1 to spontaneously insert and/or assemble into the membrane, thereby resulting in increased membrane conductance. Upon further scrutiny of the amino acid sequence of CLIC1, it was noted that it contains the motif **G**XXX**G** (at position, **G**18AKI**G**22) which is adjacent to its putative transmembrane domain and is highly conserved amongst the other members of the human CLIC family. This motif was first described to facilitate homo- and hetero-oligomerization of membrane proteins, particularly helical transmembrane domains [36] and has recently also been identified as a cholesterol binding site in the amyloid precursor protein [37]. It therefore seems likely that this motif may also be involved in CLIC1's interaction with cholesterol and may play a role in the assembly of the likely multimeric structure of CLIC1 in the membrane. This strongly supports the overall findings of the current study, where cholesterol facilitates the transition of soluble CLIC1 into membranes to form an ion channel conductive state. A further interesting observation is that a search of the CDC protein sequences, reveals that a number of the family members also contain this motif including; listeriolysin 529aa protein (**G**91YKD**G**95), perfringolysin-O 499aa protein contains 2 copies of the motif (**G**68KKA**G**72 and **G**151KVS**G**155), intermedilysin 532aa protein (**G**163LKN**G**167). This same motif is also present in the α-hemolysin 240aa protein (**G**84AST**G**88). It should also be noted that the amino acid lysine (K) is present in the CLIC1 and all of the CDC family member GXXXG motifs. The role of this motif in the membrane interactions of these proteins and their associations with cholesterol warrants further investigation.

## Conclusion

The current study demonstrates the regulatory effect of cholesterol on the ability of the spontaneously membrane inserting protein CLIC1 to interact and insert into a lipid membrane. The study suggests that CLIC1 membrane conductance arises as a result of a multi-step process involving its initial docking or binding to the membrane, then insertion into the membrane, followed by higher order quaternary arrangement of the protein into its final ion channel, conductive state. The initial steps most likely involve unfolding of the protein from its globular soluble form to a structure which exposes the hydrophobic faces of the protein as previously demonstrated under oxidative conditions [7], [16]. Cholesterol appears to be a candidate binding molecule used by CLIC1 for its initial docking onto the membrane, and it may also be required or assists in the formation of the final quaternary CLIC1 channel structure within the membrane. This is similar to the process known to exist for the cholesterol-dependent-cytolysins. However, further studies are needed to confirm this. Finally, the current study also demonstrates the feasibility of using a tethered lipid membrane system as a screening tool for the characterization of spontaneously inserting membrane and ion channel proteins.
